# 2210. Reducing Inappropriate Antibiotic Prescribing for Acute Uncomplicated Bronchitis

**DOI:** 10.1093/ofid/ofad500.1832

**Published:** 2023-11-27

**Authors:** Sabrina Williams, Rosemary M Olivero, Julie Engels, Sara Ogrin

**Affiliations:** Michigan State University College of Human Medicine, Grand Rapids, Michigan; Helen DeVos Children's Hospital of Spectrum Health, Grand Rapids, MI; Corewell Health, Hudsonville, Michigan; Helen DeVos Children's Hospital, Grand Rapids, Michigan

## Abstract

**Background:**

Antimicrobial stewardship (AS) programs aim to slow the growing threat of antimicrobial resistance. The majority of antibiotic prescribing occurs in outpatient settings. This study examines the effect of an AS intervention bundle on the proportion of inappropriate antibiotic prescriptions given to adult patients with bronchitis in outpatient settings of a large healthcare system.

**Methods:**

The Institutional Review Board approved this retrospective study of antibiotic prescription rates for adults (≥18 years old) diagnosed with bronchitis (identified using ICD-10 codes J20.9 or J20.8) during 2020 and 2021 in outpatient settings. An AS intervention bundle involving training and auditing of physicians’ antibiotic prescribing habits was enacted in January of 2021. A total of 8,176 encounters were reviewed for appropriateness of antibiotic prescriptions. Instances where no antibiotics were prescribed or antibiotics were prescribed for a comorbid condition were considered appropriate. Percentages of inappropriate antibiotic prescribing pre- and post-intervention were then compared via Chi-square analysis using SAS Enterprise Guide version 7.15 software.

**Results:**

The proportion of inappropriate antibiotic prescribing for bronchitis decreased significantly from 44.9% pre-intervention to 32.5% post-intervention (p< 0.0001). Comparison of inappropriate prescribing rates by month showed significant decreases between the pre- and post-intervention periods in March (48.1% to 31.8%, p=0.0002), October (44.4% to 30.3%, p< 0.0001), and November (42.2% to 27.0%, p< 0.0001).

**Conclusion:**

AS activities can decrease inappropriate antibiotic prescribing for bronchitis in outpatient settings. Further analyses on site-specific, seasonal and demographic differences on the effect of our AS intervention bundle may further enlighten on how programs can decrease unnecessary antibiotic use for respiratory tract infections.

**Disclosures:**

**All Authors**: No reported disclosures

